# Proteome-Wide Structural Computations Provide Insights into Empirical Amino Acid Substitution Matrices

**DOI:** 10.3390/ijms24010796

**Published:** 2023-01-02

**Authors:** Pablo Aledo, Juan Carlos Aledo

**Affiliations:** Department of Molecular Biology and Biochemistry, University of Málaga, 29071 Málaga, Spain

**Keywords:** amino acid substitution, fitness, genetic code, mutation, protein evolution, protein stability, replacement matrices, selection

## Abstract

The relative contribution of mutation and selection to the amino acid substitution rates observed in empirical matrices is unclear. Herein, we present a neutral continuous fitness-stability model, inspired by the Arrhenius law ($q_{ij}=a_{ij}e^{-\left|\Delta \Delta G_{ij}\right|}$). The model postulates that the rate of amino acid substitution (i→j) is determined by the product of a pre-exponential factor, which is influenced by the genetic code structure, and an exponential term reflecting the relative fitness of the amino acid substitutions. To assess the validity of our model, we computed changes in stability of 14,094 proteins, for which 137,073,638 in silico mutants were analyzed. These site-specific data were summarized into a 20 square matrix, whose entries, $\left|\Delta \Delta G_{ij}\right|$, were obtained after averaging through all the sites in all the proteins. We found a significant positive correlation between these energy values and the disease-causing potential of each substitution, suggesting that the exponential term accurately summarizes the fitness effect. A remarkable observation was that amino acids that were highly destabilizing when acting as the source, tended to have little effect when acting as the destination, and vice versa (source → destination). The Arrhenius model accurately reproduced the pattern of substitution rates collected in the empirical matrices, suggesting a relevant role for the genetic code structure and a tuning role for purifying selection exerted via protein stability.

## 1. Introduction

An important way in which proteins evolve is through the accumulation of amino acid changes. Spontaneous missense mutations at the DNA level are at the origin of the evolutionary process. In this regard, the rate of amino acid substitution can be directly influenced by the structure of the genetic code. That is, amino acids that can be reached by a single nucleotide mutation replace each other much more often than those that are separated by two or three nucleotides of difference between their codons. In addition, selective pressures exerted at different levels can also influence the rates of amino acid substitutions. For instance, one obvious influence comes from the impact that a given replacement has on the thermodynamic stability of the protein, which is largely determined by the extent to which the two amino acids involved in the substitution are exchangeable in terms of physicochemical properties. Another source of influence, perhaps more subtle because unrelated to protein activity, are the differences in translational efficiencies of the different codons [1], as well as the different metabolic costs of different amino acids [2,3], to name a few. 

Protein evolution, thus, is the result of a myriad of complex and interlinked processes that collectively determine the pattern of amino acid substitutions. These patterns of amino acid substitutions have been successfully summarized into global substitution matrices, denoted as ***Q***:

(1) $$Q={\left(q_{ij}\right)}_{i,j\in S},$$

where *S* is the so-called set of states, in our case formed by the 20 proteinogenic amino acids. These matrices can be empirically constructed by averaging rate substitutions over numerous sites in large sets of proteins. The first empirical ***Q*** matrix was constructed by Dayhoff and coworkers using the maximum parsimony principle [4]. This amino acid substitution matrix was later updated by Jones and colleagues, who, using the same parsimony approach, analyzed a much larger collection of protein sequences to obtain an updated matrix, known as ***Q***_JTT_ matrix [5]. Since the parsimony approach was known for underestimation of the true number of multiple amino acid substitutions on single branches of a tree, maximum likelihood inference has been successfully employed to estimate these replacement matrices. Thus, the most widely used empirical matrices, ***Q***_WAG_ [6] and ***Q***_LG_ [7], were computed using the maximum likelihood approach. 

In parallel to the development of empirical amino acid substitution matrices, the factors influencing amino acid substitution rates have received much attention [8,9,10,11]. Thus, to understand the fitness effect of mutations, two kinds of models have been proposed. The first kind of model computes the fitness as the fraction of protein found in the native state, which is a sigmoidal function of the folding free energy [12]. These models, focused on the destabilizing effect of mutations, are referred to as stability-constrained fitness models [9]. On the contrary, the second kind of model places the focus on the effect that structural changes have on fitness, regardless of the effect the structural change may have on stability. These kind of models are appropriately called structurally-constrained protein evolution models [9,10].

Many of these fitness-based models elaborate on the seminal work of Halpern and Bruno [8], who introduced a site-invariant model of codon-to-codon mutation, combined with site-specific estimates of equilibrium frequencies of each amino acid. In this way, the rates of amino acid replacement could be determined for each site, according to the effect of selection on the probability of fixation of mutations, following the Kimura equation [13]. Much more recently, following a similar approach consisting in separating the mutational process from the fixation process, Norn and coworkers proposed a stability-constrained model of protein evolution where the fitness was assumed to be proportional to the fraction of folded protein, which was determined by computing the change in thermodynamic stability (ΔΔG), using a reduced non-redundant set of 52 protein structures [11]. Despite all these efforts, the precise influence that the diverse determinants exert on the amino acid substitution rate remains an open research topic. Furthermore, none of the models previously described have been analyzed using data from a whole proteome. 

The goal of the current work was twofold. On the one hand, to generate proteomic-scale data regarding the site-specific effect of mutations on protein stability. On the other hand, to make use of these data so as to build a model of amino acid substitution in proteins that would allow assessment of the relative impact of thermodynamic stability on substitution rates; that is, to what extent the values of the empirical ***Q*** matrices are determined by the effect of the substitution on the protein stability. 

## 2. Results

### 2.1. Conservative versus Radical Amino Acid Substitution

Weber and Whelan have recently examined different criteria to classify amino acid substitutions as either conservative or radical, based on physicochemical properties of the amino acids involved [14]. They concluded that when a substitution implied a change in polarity or volume category, this was the criterion to consider the substitution radical. In every other case the substitution would be conservative. They considered this to be the best rationalization for understanding protein evolution [14]. Herein, we propose a different approach to the conservative–radical classification of amino acid substitutions, based on their effects on protein stability. 

**Figure 1:**
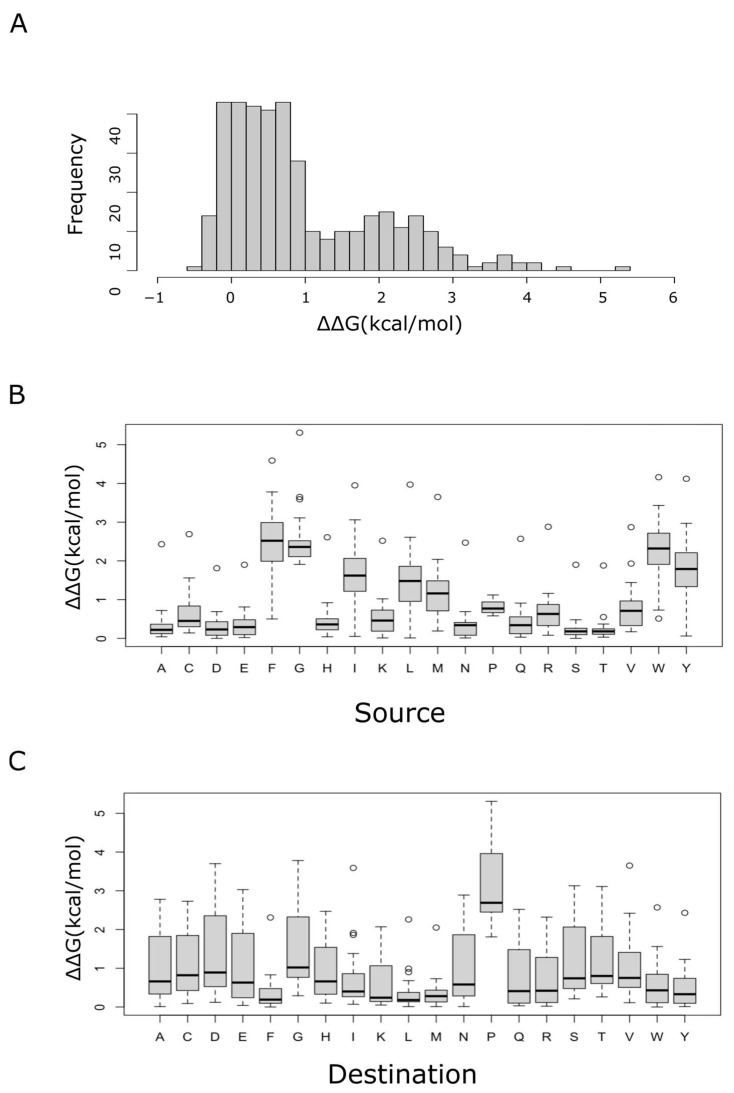
Changes in thermodynamic stability of the amino acid substitutions. For each single site in every protein from the human proteome, the thermodynamic effect (ΔΔG) of all possible amino acid substitutions was computed, which allowed us to calculate the median value for each of the 380 possible amino acid substitutions. The distribution of these median values is shown in (**A**). Box plots of these median values, depending on either the source (**B**) or the destination (**C**) amino acid, are also shown.

To this end, proteins were subjected to computational mutagenesis scans in all their positions. That is, for each single site in every protein, we computed the effect (ΔΔG) of all possible amino acid mutations using the FoldX suit [15,16]. In this way, 7,214,402 sites from 14,094 different proteins were analyzed, obtaining a 7,214,402 × 19 matrix of site-specific energy values (Table S1). All the information contained in this matrix was summarized in a 20 × 20 matrix (Table 1):

(2) $$\Delta \Delta G={\left(\Delta \Delta G_{ij}\right)}_{i,j\in S}$$

where the element corresponding to row *i* and column *j* gives the median in the energy change when an amino acid *i* (source amino acid) is substituted by an amino acid *j* (destination amino acid). A high value of the element (*i*, *j*) implied a destabilizing effect for the change of amino acid *i* to amino acid *j*. On the other hand, negative values meant stabilizing changes, which, not surprisingly, were less common (Table 1 and Figure 1).

The matrix shown in Table 1 is an asymmetric one. This asymmetry becomes evident in a very visual way when Figure 1B,C are compared. For instance, phenylalanine was a good replacement for other amino acids (little destabilizing when a destination), but it was the least readily replaced (highly destabilizing when a source). On the other hand, although proline was revealed as an amino acid always involved in radical substitutions, those changes where proline was the replacement for other residues tended to be much more destabilizing than when proline was the substituted residue. In general, Ser, Thr, Asp, Glu and Ala were amino acids most often involved in conservative substitutions when acting as sources, while those most often involved in radical substitutions were Gly, Phe, Trp, Tyr, Ile, Leu and Met. Regarding the conservative/radical nature of substitutions when focusing on the replacing (destination) amino acid, Met, Leu and Phe tended to be good replacements for most residues (conservative), while Ser, Thr, Asp, Gly and Pro were among the most radical substitutions when acting as destinations. Although these observations may point to true evolutionary trends, they must be treated with caution, since FoldX, as well as other programs based on energy functions, may sometimes overestimate the stability of hydrophobic residues, which then may appear, unrealistically, as intrinsically more stable than polar amino acids [17].

The asymmetry of the matrix indicated that, for a given amino acid, the effect on protein stability of amino acid substitutions depended on whether the amino acid was a source or a destination. Even more pertinent, the qualitative observations noted in the previous paragraph seemed to suggest an inverse effect. To quantitatively support this observation, the 20 proteinogenic amino acids were ranked from the least to the most destabilizing, either when acting as a source (x-variable), or when acting as a destination (y-variable), and a weak (R-squared = 0.22) but significant (*p*-value = 0.039) negative correlation of these ranks was then observed (Figure S1).

### 2.2. Relationship between ΔΔG and Human Diseases

**Figure 2:**
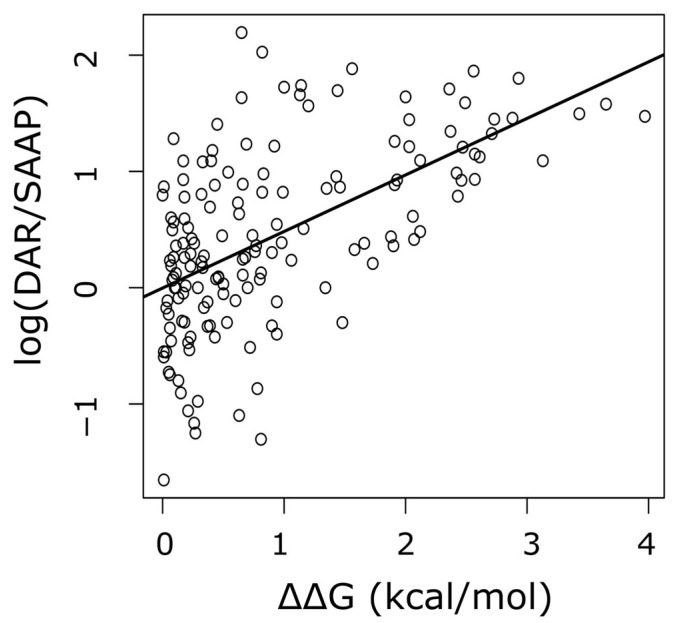
The destabilizing effect of amino acid substitution on protein structure correlates positively with the disease-causing potential.

Data collected in Table 1 represent a statistical summary of the effect of amino acid substitution on protein stability, for each of the 380 possible amino acid replacements averaged across the entire human proteome. Thus, we next took advantage of this statistical information to address the relative relevance of protein structure destabilization as the cause of diseases linked to missense mutations in humans. To this end, for each source–destination amino acid substitution we computed its disease-causing potential, defined as the number of such substitutions reported to cause disease. The disease-associated amino acid residues (DARs) [18] were divided by the number of the same source–destination substitutions observed among a collection of single amino acid polymorphisms (SAAPs) common in humans (allele frequencies above 0.01), and thought to be mostly neutral [19,20]. As observed in Figure 2, there was a very significant (*p*-value = 1.7 × 10^−14^) positive correlation between the ΔΔG values that we computed and the log of the disease-causing potential of amino acid substitutions. Thus, ΔΔG explained over 32 % of the variance in the log(DAR/SAAP).

Although this positive correlation between ΔΔG and the disease-causing potential is highly significant, its use as predictive correlation is discouraged, since its coefficient of determination is rather low (below 0.5). However, it is high enough to suggest that Table 1 may summarize the trend of the effect of each amino acid substitution on protein thermodynamic stability well. Therefore, we next built a theoretical model inspired by chemical kinetic theory, with the aim of evaluating the relevance of changes in protein stability in determining the amino acid substitution rates.

### 2.3. Arrhenius Kinetic Model for Amino Acid Substitutions

We started by considering a proteome evolving through the time according to a homogenous continuous–time Markov process. For each amino acid substitution, we had a pair of amino acids involved (i.e., $A_{i}$ and $A_{j}$) which, in a figurative sense, could be imagined as reactants of a chemical reaction:

(3) $$A_{i}⇄A_{j},$$

That is, through time amino acid $A_{i}$ can be changed to amino acid $A_{j}$, which can later be replaced again by $A_{i}$, and so on. According to the Markov theory, for a time-reversible process, such as the one we were considering, the amount of change from the state *i* to the state *j* at the equilibrium must be equal:

(4) $$\pi_{i}q_{ij}=\pi_{j}q_{ji},$$

where $\pi_{i}$ is the relative frequency of the amino acid *i* and $q_{ij}$ is the instantaneous rate of substitution of the amino acid *i* by the amino acid *j*. Equation (4), which is known as detailed balance, implies that amino acid frequencies of extant proteins are found at a steady state. Although this is a widely accepted assumption, it should be noted that some authors have pointed to the possibility that such frequencies could still be evolving [21]. Thus, following our chemical kinetic analogy, the first member of Equation (4) would be the differential rate law equation for the substitution of the state *i* by the state *j*, where $\pi_{i}$ and $q_{ij}$ play the role of concentration and rate constant, respectively. Analogously, the second member of Equation (4) would be the differential rate law for the change of amino acid *j* for amino acid *i*. Therefore, according to the Arrhenius law:

(5) $$q_{ij}=a\;e^{\frac{-E_{ij}}{b}},$$

where a is the so-called pre-exponential factor, which, in our evolutionary context, can be interpreted as the maximal absolute rate of substitution when thermodynamic stability does not exert any constraint. On the other hand, $E_{ij}$ is the activation energy for the substitution of the amino acid *i* by the amino acid *j*. The activation energy is often thought of as the magnitude of the energy barrier that, in our model, opposes the change from amino acid *i* to amino acid *j*. Since strong changes in the thermodynamic stability (ΔΔG) of a protein are evolutionarily disadvantaged, we can define:

(6) $$E={\left(E_{ij}\right)}_{i,j\in S}\;=|\Delta \Delta G|.$$

A high positive value of $\Delta \Delta G_{ij}$ implies a destabilizing effect for the change of amino acid *i* to amino acid *j*. On the other hand, negative values mean stabilizing changes, which, in terms of biological fitness, can be as detrimental as destabilizing mutations [22], particularly for metamorphic proteins [23]. Thus, *E*, the energy barrier, is understood as the obstacle that makes it difficult for a given substitution to be accepted, and was quantified as the absolute value of Table 1. Finally, b is equivalent to the product RT, used in physical chemistry as a scaling factor for energy values, since many processes and phenomena depend not on energy alone, but on the ratio of energy and RT. From now on, we refer to this parameter as “evolutionary-temperature”. 

We started by examining whether the relationship between the two variables ($q_{ij}$ and $E_{ij}$) was compatible with a negative exponential, as hypothesized (Equation (5)). Fitting the data to the linearized Arrhenius equation showed a significant linear relationship between the variables $\ln(q_{ij})$ and $E_{ij}$ (Figure S2). Despite the high statistical significance of the fit (*p*-value = 1.3 × 10^−9^), the energy barrier variable, *E*, only explained roughly 10% of the variance observed for $\ln(q_{ij})$. This high dispersion of $\ln(q_{ij})$ was not unexpected, since we were assuming a single and equal value for the parameter a, regardless of the pair of amino acids involved in the substitution. Since the value of a was interpreted as the maximal instantaneous rate of substitution, attainable in the hypothetical case that there were no thermodynamic stability constraints (*E* = 0), it seemed reasonable that different pairs of amino acids should exhibit different values of the pre-exponential factor affecting their instantaneous rate of substitution. For instance, those amino acid pairs referred to as “singlet”, i.e., those whose codons differed by just one nucleotide, were expected to present higher values of the parameter a than nonsinglet amino acid pairs. 

**Figure 3:**
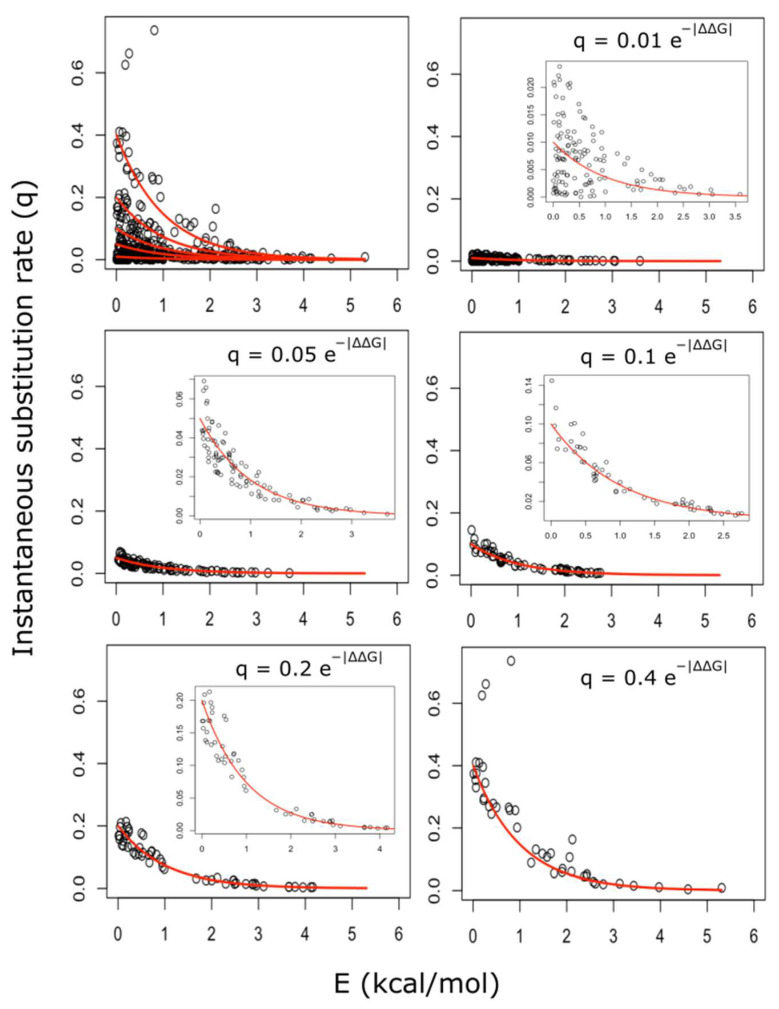
Arrhenius kinetic models accounted for the empirical instantaneous amino acid substitution rates. The top left panel shows a scatter plot *q* against *E*, where the five negative exponential curves used to partition the points are drawn in red. The remaining five panels show the indicated group resulting from the partition. The insets display the same plots, but with rescaled axes.

**Figure 4:**
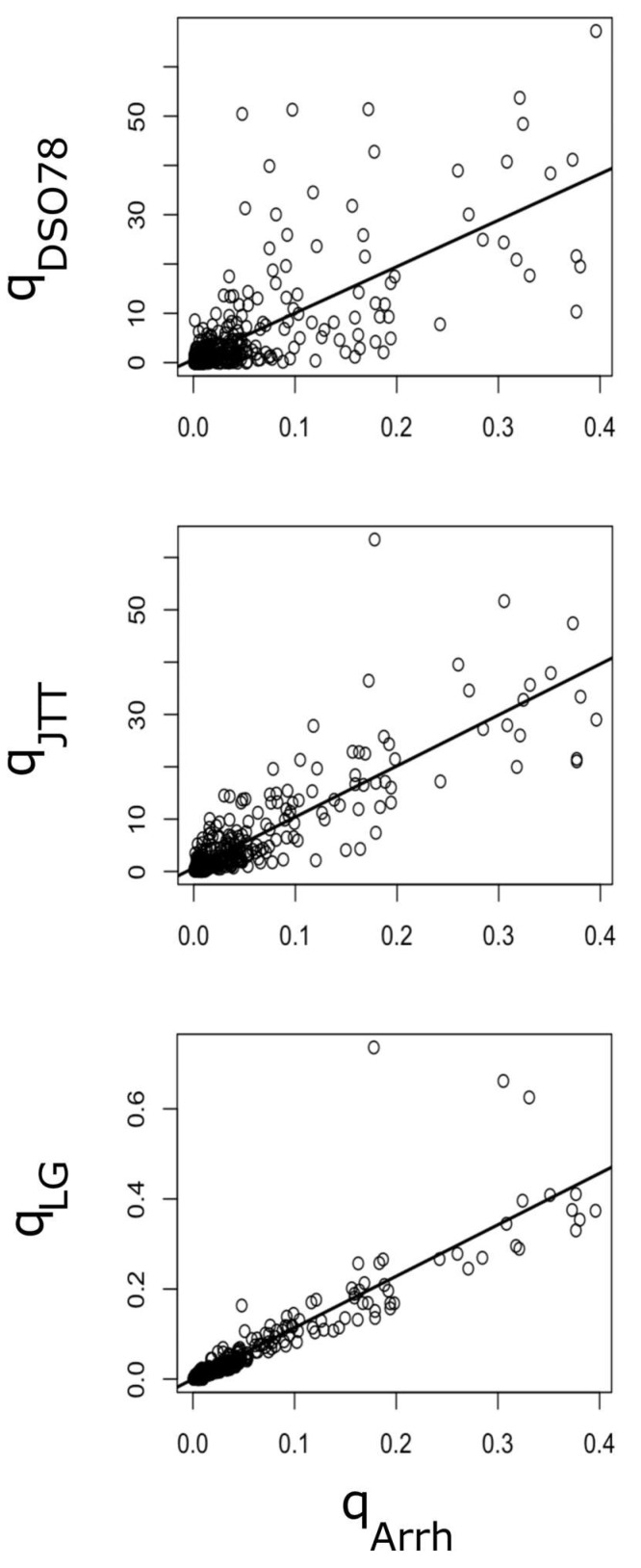
Relationship between empirical amino acid substitution matrices and the Arrhenius kinetic model. The instantaneous substitution rate values, corresponding to the ***Q***_DSO78_, ***Q***_JTT_ and ***Q***_LG_ matrices, have been plotted against the matrix computed according to the Arrhenius model. The correlation coefficients are given in Table 2, and the p-values were less than 2.2 × 10^−16^ in the three cases. The R^2^ values were 0.510, 0.730 and 0.823, respectively.

Therefore, we next considered five negative exponential curves, differing in the value of their pre-exponential factors ($a\;\in \left\{0.01,\;0.05,\;0.1,\;0.2,\;0.4\right\})$ and with evolutionary-temperature, b, constant and equal to 1. Then, the 380 possible amino acid substitutions were partitioned into five different categories of ordered pairs, according to their proximity to the five Arrhenius curves (Figure 3). To assess the degree of variation in empirical amino acid substitution matrices that could be explained by our kinetic model, we next computed, for each amino acid substitution pair, the predicted ${\hat{q}}_{ij}$ value, according to the corresponding Arrhenius kinetic model described above (Table S2). These predicted values were then compared against the empirical $q_{ij}$ values described in the literature [4,5,7,24]. As can be observed in Table 2 and Figure 4, much of the variation in empirical amino acid substitution matrices could now be explained by the Arrhenius model described above. More concretely, the Arrhenius model was able to account for 51, 73 and 82 % of the variance in the empirical instantaneous substitution rates collected into the ***Q***_DSO78_, ***Q***_JTT_ and ***Q***_LG_ matrices, respectively (Figure 4). 

### 2.4. The Structure of the Genetic Code Is a Key Determinant of the Amino Acid Substitution Rates

The Arrhenius kinetic model, that we evaluated in the previous section, distinguished five groups of amino acid substitutions (Table S2), which differed in their pre-exponential factor values (parameter a from Equation (5)). Since this parameter could be interpreted as the maximal absolute rate of substitution when protein stability did not exert any constraint, it seemed reasonable to postulate a relevant role for the structure and configuration of the standard genetic code as determinant of the exponential factor values. To address such a hypothesis, and shed some light on what features of the genetic code could be more relevant in determining the correct assignment of the pre-exponential factor in our Arrhenius kinetic model, we next resorted to machine learning. 

**Figure 5:**
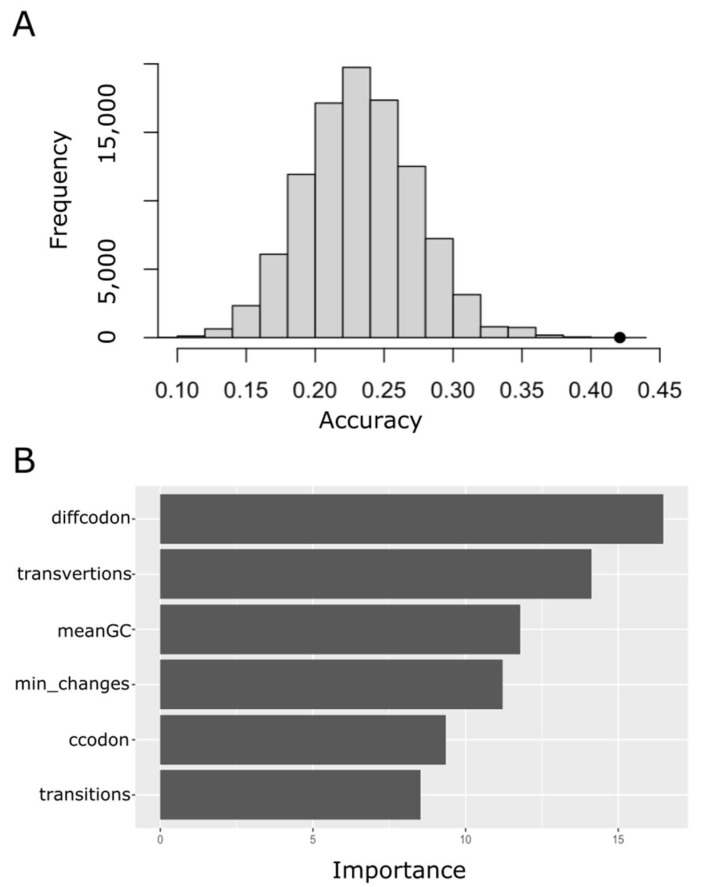
Performance of the predictors related to the genetic code employing a random forest classifier. (**A**) Null distribution for the accuracy. To build this distribution a random classifier acted on 100,000 samples with the same size and data strata as the testing set. The filled black circle points to the accuracy for the random forest classifier on the testing set. (**B**) Variable importance plot for the random forest classifier. The method used to compute variable importance was the mean decrease in node impurity (Gini importance).

Thus, different supervised multiclass classification techniques were implemented and evaluated on a 5-fold cross validation. Table 3 shows the performance of these predictive models in terms of accuracy, sensitivity, specificity and area under the receiver operating characteristic curve (AUROC). Random forests provided the best performance. Nevertheless, in terms of accuracy, all the assayed models performed significantly better than a random classifier. Figure 5A shows the accuracy distribution for random guesses on 100,000 samples of the same size as the testing set and with the same class strata. As expected, the random accuracy was normally distributed with a mean of 0.234 and standard deviation of 0.041. The much higher accuracy of the random forest classifier (0.421 ± 0.025, mean ± sd) suggested that the structure of the genetic code indeed played a relevant role in determining the amino acid substitution groups. To further gain an understanding of what characteristics of the genetic code were the ones that had the greatest weight when determining the substitution group, we built a variable importance plot for the random forest classifier. As is shown in Figure 5B, the difference in the number of triplets coding for the source and destination amino acids, and the mean number of transversions involved in the codon substitution, were the main predictors for the random forest model. 

## 3. Discussion

Complex patterns of amino acid substitution developed during protein evolution. These patterns are captured in the so-called empirical substitution matrices. Since these matrices reflect the outcome of the forces that collectively control the patterns of amino acid substitutions, they have been invaluable to detect homologs [25], reconstruct phylogenies [7], infer the primary structure of ancestral forms [26], trace the co-evolution of different positions within a protein [27], etc. Although in recent years there have been remarkable advances in the development of alignment-free sequence comparison methods to address protein evolution [28,29], the mainstream approaches in the field are still based on sequence alignment methods that make extensive use of substitution matrices [30]. Nevertheless, despite the great practical utility of these matrices in bioinformatics and evolutionary biology, they fail to provide insight into the mechanisms shaping the global pattern of amino acid substitution encapsulated within them. Thus, we still do not know the relative importance of the selective pressures acting on the biophysical properties of proteins, on the one hand, and the structure of the genetic code and mutational biases, on the other [9,14]. 

Work carried out over the last decade has shown, in the context of protein evolution, the usefulness of introducing the perspective of thermodynamic stability [31,32,33,34]. However, none of these studies addressed the effect of mutations on protein stability at a proteomic scale. Thus, to what extent stability shapes protein evolution and determines the observed amino acid substitution rates, remains an open question [9]. Since global substitution matrices are constructed by averaging rate values observed over numerous sites in many proteins, unraveling the relationship between these rates and changes in stability must require averaging ∆∆G across many sites in many proteins as well. In a promising recent work, Norn and coworkers, using the Rosetta modeling suite, addressed the thermodynamic effect (∆∆G) of all the possible amino acid mutations in 52 protein structures [11]. Herein, using super-computational resources and taking advantage of the AlphaFold project [35,36], we assessed the changes in stability (∆∆G) for all possible mutations at all sites in 14,094 different human proteins. To the best of our knowledge, this is the first work to report on site-specific changes in stability on an entire proteome (Table S1). In order to use all this wealth of data in a model that may provide insights on the relative importance of stability constraints, site-specific ∆∆G values were averaged over sites and proteins (Table 1). The significant positive correlation observed between the median ∆∆G and the disease-causing potential (Figure 2), indicated that these ∆∆G values acceptably summarized the effect of the considered substitutions on protein stability, and reinforced the idea that mutations leading to extreme values of ∆∆G are preferential targets of purifying selection. In this way, ∆∆G can be envisioned as an energy barrier that opposes the fixation of the considered mutation. This view is reminiscent of a well-known kinetic function, namely, the Arrhenius law (see Equation (5)). Indeed, the values of the ***Q*** matrices are instantaneous rates, making the proposed kinetic analogy appropriate. Nevertheless, it should be pointed out that we are not claiming that the amino acid substitution rates behave according to the kinetic theory of collisions. That is, the Arrhenius analogy we present herein is just that, an analogy. In any case, this analogy can be useful, as we discuss shortly, in dissecting the relative contribution of mutational rates and purifying selection. However, a brief consideration of the Arrhenius law is necessary.

The Arrhenius equation gives the rate constant, *k*, of a chemical reaction as the product of a pre-exponential (“frequency”) factor *A* and an exponential term:

(7) $$k=A\;e^{\frac{-E_{a}}{RT}},$$

where *R* is the gas constant and *E_a_* is the so-called activation energy, there is an energy barrier opposing the conversion of reagents into products. In physical chemistry, this equation is used to characterize the temperature dependence of reaction rates. Temperature affects both the exponential term, as well as the pre-exponential factor [37]. Indeed, using the transition state theory, introduced by Henry Eyring [38], it can be shown that:

(8) $$A=\frac{k_{\beta }}{h}ee^{\frac{\Delta S^{‡}}{R}}T,$$

However, often *A* is treated as temperature independent [39]. The justification of this widespread practice boils down to the fact that temperature has a much greater impact on the exponential term than on the pre-exponential factor. Now, going back to our model on instantaneous substitution rates, we started by assuming that the main contribution to the variance of $q_{ij}$ was due to the exponential term (variable $E_{ij}$), while keeping a constant pre-exponential factor, equal for all the amino acid substitution pairs (Equation (5)). That is:

(9) $$a_{ij}\approx a,\;\;\;\;\;\forall \;i,\;j\in S,$$

In a way, this working hypothesis assumes that the role of selection (at the protein level) is more of a determinant than the role of mutations (at the DNA level). However, such a model could only explain around 10% of the $q_{ij}$ variance (Figure S2). In contrast, when the pre-exponential factor was allowed to vary, most of the variance of $q_{ij}$ could be satisfactorily explained by the variable $E_{ij}$ (Figure 3). These results suggested that the overall instantaneous rate of substitution, $q_{ij}$ could be expressed as the product of the following two factors: one, represented by the pre-exponential term $\left(a_{ij}\right)$, which collects all the terms affecting the rates of codon mutations leading from *i* to *j*, and the other term, $e^{-E_{ij}}$, takes values between 0 and 1, and can be envisioned as the probability of the indicated (i→j) codon mutation going to fixation. That is, the exponential term captures the relative fitness effect of this amino acid substitution. In this respect, our kinetic Arrhenius model represents a neutral continuous fitness-stability model able to explain empirical amino acid substitution matrices (Figure 4), and, therefore, useful to provide insight into the evolutionary processes shaping the complex pattern of observed amino acid substitutions [4,5,7]. In this sense, our results not only suggest a prominent role for genetic code configuration and a tuning role for the stability effect via selection, but also point to the *diffcodon* and *transversion* (see Section 4 for definitions) as the most relevant features influencing the substitution rate through their effects on the pre-exponential term (Figure 5). 

The fact that empirical matrices have been so successful in practice, for evolutionary biologists and bioinformaticians, should encourage us to search for mechanistic models able to reproduce the empirical data and explain, in terms of evolutionary processes, the origins of the underlying patterns. The kinetic Arrhenius model we present herein is a neutral continuous fitness-stability model that seems to fulfil these conditions.

It should be noted that the Arrhenius model proposed herein is, regarding the mathematical relationship between variables, not very different from the Kimura equation for the transition probability [13] and its interpretation in terms of a stationary Markov process [8,40]. Thus, the current work further supports, on a proteomic scale, the sigmoidal relationship between variables previously proposed. The models based on Kimura’s formula have been in use for a long time, and they are based on selection mechanisms widely accepted in population genetics. However, all these models rest on the premise that the fitness of an organism carrying a mutation in a protein is a function of the fraction of that protein that is folded [11]. Thus, although the idea is appealing, experimental evidence supporting the validity of such a premise is rather scant. Since correlation does not imply causation, further work is still required to firmly establish the biological mechanisms underlying the relationship between instantaneous rates of substitution and protein stability. In this respect, the accompanying site-specific data regarding the stability of over 137,000,000 in silico mutants aimed to be of help. 

## 4. Materials and Methods

### 4.1. Protein Dataset

The human reference proteome was obtained from UniProt [41]. From the set formed by the canonical representative peptide of each gene, those with molecular sizes either below 100 or above 2670 residues were filtered out. This selection finally yielded a dataset consisting of 7,214,402 residues from 14,094 different proteins. For each of these proteins, a file with protein data bank (pdb) format was obtained from the AlphaFold protein structure database [35,42]. 

### 4.2. Thermodynamic Stability Changes

Each of the 7,214,402 amino acids found in 14,094 proteins of our dataset, was mutated to 19 complementary amino acids and the effect of these substitutions on protein stability (ΔΔG) was computed using the force-field FoldX 5.0 [16], as implemented by *ptm*, an R package designed to assist in the study of proteins [42,43]. All these computations were carried out at the Supercomputing and Bioinnovation Center of the University of Malaga (https://www.scbi.uma.es/site, accessed on 12 October 2022). 

### 4.3. Estimation of Substitution Rates According to the Arrhenius Kinetic Model

A scatter plot showing the relationship between the energy barrier we computed, $\Delta \Delta G_{ij}$, and the empirical substitution rate, $q_{ij}$, was built. Afterwards, we considered five negative exponential curves, differing in the value of their pre-exponential factors ($a\;\in \left\{0.01,\;0.05,\;0.1,\;0.2,\;0.4\right\})$ and with evolutionary-temperature, b, constant and equal to 1. Each of the 380 possible amino acid substitutions was partitioned into five different categories according to their proximity to the five Arrhenius curves (Figure 3). In this way, for each amino acid pair, we could compute an estimated instantaneous substitution rate, ${\hat{q}}_{ij},$ using the energy barrier values from Table 1 and Equation (5). In matrix form, these substitution rate values are denoted as:

(10) $$Q_{Arrh}={\left({\hat{q}}_{ij}\right)}_{i,j\in S},$$

### 4.4. Correlation between Empirical Amino Acid Substitution Matrices

The exchangeability matrices, ***S***, linked to the substitution matrices, ***Q***, reported by Dayhoff and coworkers [4] (DSO78), Jones and coworkers [5] (JTT) and Le and Gascuel [7] (LG), were obtained from the PAML 4.8 package [44]. More specifically, the data files ‘dayhoff.dat’, ‘jones.dat’ and ‘lg.dat’, provided by PAML, were read and used to compute ***Q***_DSO78_, ***Q***_JTT_ and ***Q***_LG_, respectively. To this end, we used the matricial equality Q=S Π, where Π is a diagonal matrix containing the stationary amino acid frequencies, also provided by PAML 4.8 in the referred data files. The instantaneous substitution rates predicted by the Arrhenius kinetic model, ***Q***_Arrh_, were taken as the independent variables while the empirical substitution rates described above were taken as the dependent variables. Regression analyses were conducted using standard R functions. 

### 4.5. Machine Learning

For each of the 380 substitution pairs ($aa_{i}$ *→* $aa_{j}$), we extracted the following features, all of which are related to the genetic code structure:

- *diffcodon*: the number of triplets coding for $aa_{j}$ minus the number of triplets coding for $aa_{i}$.
- *ccodon*: the number of triplets coding for $aa_{j}$ times the number of triplets coding for $aa_{i}$.
- *transitions*: number of transitions counted among the *ccodon* triplet pairs, divided by the total number of nucleotide changes.
- *transversions*: number of transversions counted among the *ccodon* triplet pairs, divided by the total number of nucleotide changes.
- *min_changes*: minimum number of nucleotide changes allowing the substitution being considered. That is, among the *ccodon* triplet pairs the one exhibiting the minimum number of changes is selected and that number taken.
- *min_transitions*: as above, but limited to transition changes.
- *min_transvertions*: as above, but referred to as transversion changes.
- *meanGC*: mean difference in the GC content of codons (codons for $aa_{j}$—codons for $aa_{i})$.
- *minGC*: minimal difference in the GC content of codons (codons for $aa_{j}$—codons for $aa_{i})$.
- *maxGC*: maximum difference in the GC content of codons (codons for $aa_{j}$—codons for $aa_{i})$.

All the subsequent steps, described below, were executed within the framework of ‘tidymodels’, which consists of a collection of R packages for modeling and machine learning [45]. Briefly, after controlling for correlation between features and standardizing their values, data were randomly partitioned into five-fold for posterior cross-validation. We trained k-nearest neighbor (k-NN), decision tree (DT), bagging tree (BG), random forest (RF) and boost tree (BT) models, using the following engines: kknn, rpart, rpart, ranger and xgboost, respectively. For model tuning, the parameters used for each model were optimized using random grids of size 27 and the AUROC as metrics. The performance of all the models was evaluated on the five-fold cross-validation sets using accuracy, sensitivity, specificity and AUROC as metrics, as described in [46]. The R script used to implement these steps can be obtained at https://bitbucket.org/jcaledo/qarrheniu s/src/master/Scripts (accessed on 12 October 2022).

## Figures and Tables

**Table 1:** Median for substitution of the source amino acid (rows) for the destination amino acid (columns) in kcal/mol. Substitutions are labelled as radical, according to the kcal/mol criterion, but as conservative when the polarity-volume criterion is used, shown in green. Those considered as radical, regardless of the criterion used, are shown in red. On the other hand, substitutions classified as conservative by both criteria are shown in blue. Finally, substitutions considered conservative by the energy criterion, but as radical when the polarity–volume criterion was used, are indicated in purple.

	A	R	N	D	C	Q	E	G	H	I	L	K	M	F	P	S	T	W	Y	V
A	.	−0.12	0.15	0.41	0.32	−0.07	0.09	0.72	0.25	0.28	−0.16	−0.15	−0.30	−0.12	2.43	0.22	0.53	0.04	−0.05	0.50
R	0.66	.	0.58	0.97	0.82	0.33	0.63	1.16	0.66	0.60	0.17	0.13	0.08	0.24	2.88	0.74	0.94	0.43	0.33	0.93
N	0.35	−0.06	.	0.34	0.42	0.08	0.25	0.67	0.34	0.40	−0.08	−0.08	−0.17	−0.07	2.47	0.39	0.66	0.16	0.01	0.69
D	0.32	0.12	0.17	.	0.43	0.12	0.07	0.62	0.32	0.43	0.01	0.05	−0.07	0.00	1.81	0.42	0.65	0.23	0.09	0.69
C	0.20	0.45	0.36	0.89	.	0.41	0.78	1.14	0.61	0.31	−0.14	0.22	−0.28	0.65	2.69	0.54	0.45	1.56	1.00	0.29
Q	0.38	−0.03	0.34	0.62	0.53	.	0.23	0.91	0.37	0.20	−0.13	−0.06	−0.29	−0.05	2.57	0.49	0.63	0.11	0.05	0.58
E	0.29	0.02	0.34	0.43	0.53	0.03	.	0.81	0.29	0.17	−0.18	−0.07	−0.27	−0.12	1.90	0.47	0.57	0.05	−0.03	0.49
G	2.12	1.91	2.10	2.37	2.36	2.31	2.47	.	2.47	3.59	2.26	2.04	2.05	2.31	5.31	2.06	3.11	2.57	2.43	3.65
H	0.36	−0.11	0.23	0.66	0.42	0.04	0.32	0.92	.	0.21	−0.33	−0.18	−0.50	−0.39	2.61	0.48	0.58	−0.11	−0.23	0.51
I	2.02	1.43	2.03	2.80	1.88	1.61	2.10	3.06	1.62	.	0.06	1.20	0.05	0.83	3.95	2.49	1.73	1.54	1.23	0.81
L	1.67	1.13	1.73	2.34	1.81	1.35	1.70	2.61	1.46	0.78	.	0.93	−0.01	0.44	3.97	2.07	1.91	0.98	0.77	1.48
K	0.50	0.05	0.46	0.79	0.71	0.21	0.46	1.02	0.51	0.21	0.01	.	−0.09	0.07	2.52	0.62	0.80	0.26	0.16	0.75
M	1.24	0.82	1.27	1.68	1.49	0.95	1.16	2.04	1.16	0.76	0.19	0.65	.	0.42	3.65	1.48	1.58	0.66	0.63	1.34
F	2.78	2.32	2.89	3.70	2.73	2.52	3.03	3.78	2.30	1.91	0.90	2.07	0.63	.	4.59	3.13	2.95	0.94	0.50	2.42
P	0.90	0.63	0.90	0.80	1.00	0.70	0.58	1.02	0.77	0.94	0.68	0.65	0.64	0.64	.	0.94	1.06	0.75	0.71	1.12
S	−0.01	−0.24	−0.01	0.12	0.09	−0.15	−0.04	0.29	0.10	0.26	−0.18	−0.24	−0.29	−0.18	1.90	.	0.26	0.00	−0.11	0.48
T	0.15	−0.18	0.11	0.31	0.20	−0.08	0.06	0.55	0.11	−0.07	−0.28	−0.21	−0.37	−0.19	1.88	0.21	.	0.03	−0.10	0.11
W	2.49	2.12	2.64	3.24	2.56	2.32	2.67	3.43	2.13	1.86	0.99	1.96	0.73	0.51	4.16	2.93	2.76	.	0.82	2..30
Y	1.97	1.53	2.00	2.71	2.03	1.77	2.15	2.97	1.66	1.38	0.42	1.29	0.26	−0.06	4.12	2.46	2.27	0.45	.	1.79
V	0.94	0.42	0.99	1.44	0.91	0.60	0.92	1.93	0.73	−0.27	−0.18	0.28	−0.17	0.17	2.87	1.37	0.71	0.61	0.38	.

**Table 2:** Correlation matrix for different ***Q***-matrices. The table displays the correlation coefficients for different empirical matrices and the matrix computed using the Arrhenius kinetic model presented in the current work (***Q***_Arrh_).

	*Q*_DSO78_	*Q*_JTT_	*Q*_LG_	*Q*_Arrh_
***Q***_DSO78_	1			
***Q***_JTT_	0.7898	1		
***Q***_LG_	0.7065	0.9166	1	
***Q***_Arrh_	0.7122	0.8533	0.9073	1

**Table 3:** Supervised multiclass classification of amino acids substitutions. A 5-fold cross-validation approach was employed to evaluate the performance of 5 models, using as metric the accuracy, sensitivity, specificity and area under the receiver operating characteristic curve (AUROC). The *p*-value column indicates the probability of obtaining an accuracy equal to, or higher than, that of the corresponding model, when the predictions were made randomly on a dataset with the same size and structure as the testing set.

Model	Accuracy	Sensitivity	Specificity	AUROC	*p*-Value
k-NN (k = 1)	0.375 ± 0.035	0.312 ± 0.031	0.835 ± 0.009	0.570 ± 0.019	0.00059
Decision trees	0.391 ± 0.038	0.331 ± 0.038	0.839 ± 0.009	0.665 ± 0.027	0.00018
Bagging trees	0.391 ± 0.032	0.327 ± 0.023	0.842 ± 0.008	0.671 ± 0.013	0.00018
Random forests	0.421 ± 0.025	0.357 ± 0.026	0.849 ± 0.005	0.682 ± 0.012	0.00001
Boost trees	0.385 ± 0.019	0.315 ± 0.023	0.838 ± 0.005	0.662 ± 0.016	0.00018

## Data Availability

The data used in this study are available at https://bitbucket.org/jcaledo/qarrhenius/src/master/Data (accessed on 12 October 2022).

## Supplementary Materials

The following are available online at https://www.mdpi.com/article/10.3390/ijms24010796/s1.
