# Supplementary material for: Proteome-Wide Structural Computations Provide Insights into Empirical Amino Acid Substitution Matrices
Source: Int J Mol Sci. 2023 Jan 2;24(1):796. doi: 10.3390/ijms24010796 (PMC9821064; doi:10.3390/ijms24010796)
Supplement: Supplementary file 1 [file ijms-24-00796-s001.zip › ijms-2050031-supplementary.pdf]

Supplementary Material

Proteome-wide structural computations provide insights into empirical amino acid substitution matrices

Pablo Aledo and Juan C. Aledo*

Department of Molecular Biology and Biochemistry, University of Málaga, 29071-Málaga, Spain.

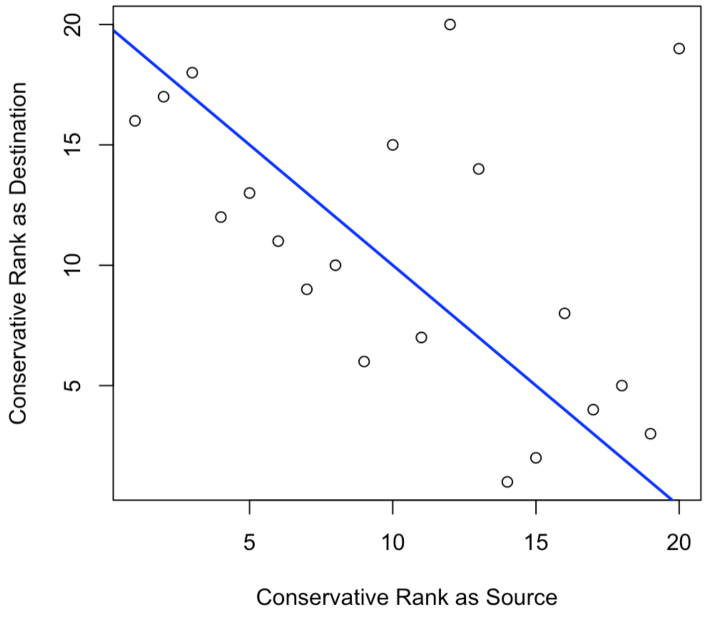

Fig. S1. Conservative ranks were computed as follows. Each amino acid, when considered either as source or as destination, received a number from 1 to 20. A value of 1 means this amino acid averages the lowest $\Delta\Delta G$. On the contrary, scoring 20 means the more radical source or destination on average, with the highest $\Delta\Delta G$. The blue line shows the line for a hypothetical perfect negative correlation of ranks (y = -x).

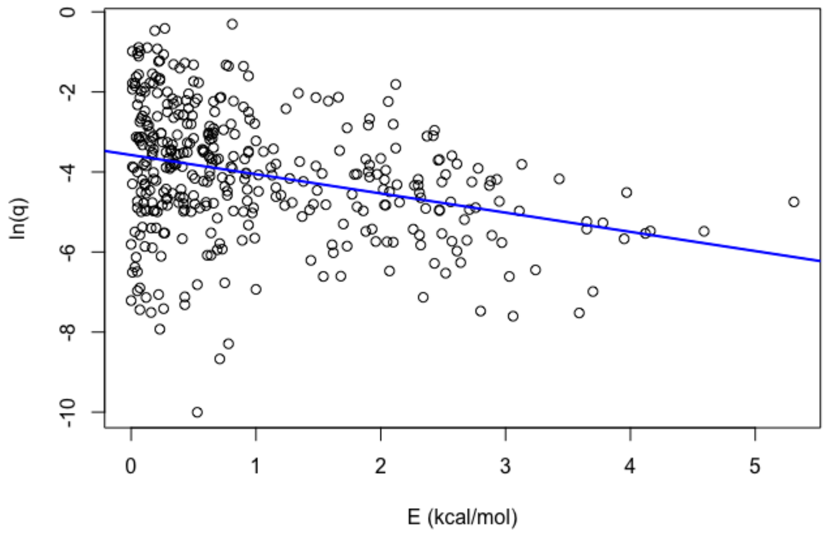

Fig. S2. The linearized Arrhenius equation was used to carry out linear regression of ln(q) on E. The dispersion plot for the 380 points and the fitted regression line (in blue) are shown. The R-squared was 0.093 and the p-value = 1.3 10^{-9}.

Table S1. Site-specific stability changes, $\Delta\Delta G$, due to mutations in human proteins. Values are shown as kcal/mol. The table can be obtained as an Rdata at:

https://bitbucket.org/jcaledo/qarrhenius/src/master/TableS1.Rda.

Table S2. Extracted features used in supervised learning and predicted values for $\boldsymbol{Q}_{\mathrm{Arrh}}$. The table can be obtained as an Rdata at:

https://bitbucket.org/jcaledo/qarrhenius/src/master/TableS2.Rda

Table S3. Measures of central tendency and dispersion for changes in thermodynamic stability of the amino acid substitutions. N stands for the number of the indicated mutation analyzed. Min and Max provide the range of energies observed. Median, mean and standard deviation (SD) are given in kcal/mol.

Mutation	N	Min	Median	Mean	Max	SD
A -> R	509,804	-5.23	-0.12	1.88	53.88	5.22
A -> N	509,804	-3.68	0.15	0.96	20.19	2.20
A -> D	509,804	-6.4	0.41	1.41	21.14	2.58
A -> C	509,804	-5.53	0.32	0.50	10.32	0.86
A -> Q	509,804	-3.32	-0.07	1.12	43.38	3.02
A -> E	509,804	-6.12	0.09	1.39	42.53	3.25
A -> G	509,804	-8.00	0.72	0.61	5.42	1.05
A -> H	509,804	-3.36	0.25	0.96	83.98	2.40
A -> I	509,804	-4.99	0.28	1.05	41.80	2.14
A -> L	509,804	-4.84	-0.16	0.61	27.2	2.43
A -> K	509,804	-4.72	-0.15	1.28	44.96	3.69
A -> M	509,804	-5.36	-0.30	0.27	33.83	2.19
A -> F	509,804	-5.29	-0.12	2.86	75.94	7.33
A -> P	509,804	-5.67	2.43	2.26	27.40	2.49
A -> S	509,804	-3.27	0.22	0.33	9.94	0.72
A -> T	509,804	-3.03	0.53	0.86	29.08	1.24
A -> W	509,804	-5.10	0.04	4.74	89.77	10.99
A -> Y	509,804	-4.78	-0.05	3.61	74.28	8.71
A -> V	509,804	-4.22	0.50	0.90	34.07	1.36

Mutation	N	Min	Median	Mean	Max	SD
R -> A	426,630	-14.79	0.66	0.85	9.77	0.94
R -> N	426,630	-8.17	0.58	0.81	14.52	1.04
R -> D	426,630	-10.71	0.97	1.37	16.53	1.61
R -> C	426,630	-14.93	0.82	1.01	9.41	0.88
R -> Q	426,630	-4.93	0.33	0.58	17.89	0.93
R -> E	426,630	-7.52	0.63	0.99	17.24	1.42
R -> G	426,630	-19.19	1.16	1.32	11.53	1.30
R -> H	426,630	-7.24	0.66	0.77	65.80	1.13
R -> I	426,630	-6.24	0.60	0.87	35.47	1.21
R -> L	426,630	-8.37	0.17	0.24	21.14	0.85
R -> K	426,630	-5.39	0.13	0.29	18.23	0.72
R -> M	426,630	-9.65	0.08	0.12	14.90	0.83
R -> F	426,630	-7.24	0.24	0.68	74.45	2.10
R -> P	426,630	-8.11	2.88	2.75	24.31	2.42
R -> S	426,630	-14.14	0.74	0.98	11.13	1.05
R -> T	426,630	-6.86	0.94	1.17	28.45	1.08
R -> W	426,630	-4.66	0.43	1.03	63.13	2.57

R -> Y	426,630	-7.26	0.33	0.91	56.75	2.65
R -> V	426,630	-6.32	0.93	1.18	29.68	1.07

**Mutation**	**N**	**Min**	**Median**	**Mean**	**Max**	**SD**
N -> A	256,297	-8.03	0.35	0.51	8.13	0.84
N -> R	256,297	-4.52	-0.06	0.44	52.45	2.16
N -> D	256,297	-6.22	0.34	0.53	12.34	1.15
N -> C	256,297	-8.92	0.42	0.59	8.53	0.75
N -> Q	256,297	-3.57	0.08	0.40	14.73	1.20
N -> E	256,297	-4.31	0.25	0.59	18.14	1.42
N -> G	256,297	-15.30	0.67	0.79	9.19	1.14
N -> H	256,297	-3.38	0.34	0.45	56.25	1.10
N -> I	256,297	-5.50	0.40	0.77	24.29	1.47
N -> L	256,297	-6.00	-0.08	0.016	19.28	1.05
N -> K	256,297	-5.11	-0.08	0.27	25.92	1.46
N -> M	256,297	-5.30	-0.17	-0.08	27.40	1.10
N -> F	256,297	-5.42	-0.07	0.65	63.82	3.20
N -> P	256,297	-5.64	2.47	2.37	21.23	2.39
N -> S	256,297	-7.22	0.39	0.61	8.13	0.91
N -> T	256,297	-5.45	0.66	0.91	18.12	1.07
N -> W	256,297	-5.32	0.16	1.42	73.36	5.00
N -> Y	256,297	-4.93	0.01	1.01	57.55	4.18
N -> V	256,297	-5.57	0.69	0.97	23.16	1.19

**Mutation**	**N**	**Min**	**Median**	**Mean**	**Max**	**SD**
D -> A	350,568	-11.91	0.32	0.65	10.84	1.31
D -> R	350,568	-11.15	0.12	0.73	36.83	2.30
D -> N	350,568	-9.07	0.17	0.43	10.34	1.10
D -> C	350,568	-8.29	0.43	0.78	9.95	1.24
D -> Q	350,568	-6.74	0.12	0.58	18.44	1.58
D -> E	350,568	-5.16	0.07	0.43	16.68	1.24
D -> G	350,568	-15.92	0.62	0.98	11.78	1.45
D -> H	350,568	-9.00	0.32	0.63	69.80	1.40
D -> I	350,568	-7.67	0.43	0.84	33.69	1.63
D -> L	350,568	-7.86	0.01	0.32	25.97	1.52
D -> K	350,568	-9.25	0.05	0.57	32.87	1.85
D -> M	350,568	-9.00	-0.07	0.17	15.89	1.49
D -> F	350,568	-8.43	0.00	0.71	54.48	2.94
D -> P	350,568	-8.37	1.81	1.98	26.11	2.40
D -> S	350,568	-10.20	0.42	0.77	10.94	1.30
D -> T	350,568	-7.64	0.65	1.00	21.29	1.37
D -> W	350,568	-6.43	0.23	1.29	69.86	4.22
D -> Y	350,568	-7.50	0.09	0.96	57.86	3.52
D -> V	350,568	-8.49	0.69	1.04	25.03	1.44

**Mutation**	**N**	**Min**	**Median**	**Mean**	**Max**	**SD**
C -> A	162,226	-8.42	0.20	0.63	9.12	1.52

C -> R	162,226	-4.57	0.45	3.23	46.88	5.61
C -> N	162,226	-3.79	0.36	1.29	25.02	2.56
C -> D	162,226	-7.54	0.89	1.86	23.12	2.92
C -> Q	162,226	-4.50	0.41	1.85	28.48	3.30
C -> E	162,226	-5.17	0.78	2.16	27.51	3.41
C -> G	162,226	-13.00	1.14	1.39	8.62	1.68
C -> H	162,226	-3.34	0.61	1.51	87.73	2.73
C -> I	162,226	-4.28	0.31	1.26	25.04	2.80
C -> L	162,226	-5.34	-0.14	0.79	24.69	2.98
C -> K	162,226	-5.02	0.22	2.18	36.51	4.03
C -> M	162,226	-4.91	-0.28	0.53	31.52	2.81
C -> F	162,226	-5.23	0.65	4.27	65.69	7.13
C -> P	162,226	-4.95	2.69	2.85	25.44	2.72
C -> S	162,226	-6.71	0.54	1.11	10.43	1.82
C -> T	162,226	-3.58	0.45	1.24	16.88	2.24
C -> W	162,226	-5.80	1.56	7.43	77.58	11.24
C -> Y	162,226	-4.61	1.00	5.74	72.00	8.96
C -> V	162,226	-3.71	0.29	0.99	18.65	2.23

**Mutation**	**N**	**Min**	**Median**	**Mean**	**Max**	**SD**
Q -> A	362,053	-29.00	0.38	0.40	5.66	0.54
Q -> R	362,053	-10.44	-0.03	0.05	32.06	1.00
Q -> N	362,053	-11.01	0.34	0.38	11.44	0.68
Q -> D	362,053	-11.98	0.62	0.71	10.66	0.98
Q -> C	362,053	-23.87	0.53	0.58	5.10	0.54
Q -> E	362,053	-9.49	0.23	0.30	13.96	0.67
Q -> G	362,053	-39.74	0.91	0.86	7.05	1.01
Q -> H	362,053	-11.01	0.37	0.30	58.96	0.62
Q -> I	362,053	-4.92	0.20	0.32	40.45	0.92
Q -> L	362,053	-6.18	-0.13	-0.23	16.00	0.65
Q -> K	362,053	-10.09	-0.06	-0.04	17.63	0.59
Q -> M	362,053	-17.22	-0.29	-0.35	11.50	0.69
Q -> F	362,053	-4.73	-0.05	0.18	52.94	1.71
Q -> P	362,053	-30.00	2.57	2.31	22.94	2.10
Q -> S	362,053	-24.65	0.49	0.54	6.69	0.68
Q -> T	362,053	-14.30	0.63	0.71	30.72	0.74
Q -> W	362,053	-5.06	0.11	0.54	61.16	2.47
Q -> Y	362,053	-4.41	0.05	0.42	52.87	2.27
Q -> V	362,053	-14.12	0.58	0.68	33.24	0.78

**Mutation**	**N**	**Min**	**Median**	**Mean**	**Max**	**SD**
E -> A	546,081	-17.42	0.29	0.39	8.16	0.74
E -> R	546,081	-8.65	0.02	0.20	40.46	1.31
E -> N	546,081	-10.36	0.34	0.42	10.82	0.88
E -> D	546,081	-8.85	0.43	0.47	10.98	0.78
E -> C	546,081	-15.65	0.53	0.61	8.90	0.77
E -> Q	546,081	-7.14	0.03	0.11	13.67	0.68

E -> G	546,081	-33.27	0.81	0.83	9.81	1.12
E -> H	546,081	-7.48	0.29	0.31	56.00	0.76
E -> I	546,081	-7.66	0.17	0.31	23.60	1.00
E -> L	546,081	-8.56	-0.18	-0.15	16.87	0.79
E -> K	546,081	-9.79	-0.07	0.11	22.53	1.02
E -> M	546,081	-8.65	-0.27	-0.25	12.80	0.84
E -> F	546,081	-7.30	-0.12	0.11	50.50	1.54
E -> P	546,081	-10.15	1.90	1.89	27.34	2.08
E -> S	546,081	-16.40	0.47	0.58	8.72	0.89
E -> T	546,081	-13.04	0.57	0.69	19.00	0.88
E -> W	546,081	-5.46	0.05	0.42	61.02	2.19
E -> Y	546,081	-7.12	-0.03	0.30	44.66	1.92
E -> V	546,081	-10.32	0.49	0.66	37.89	0.89

**Mutation**	**N**	**Min**	**Median**	**Mean**	**Max**	**SD**
G -> A	479,056	-2.82	2.12	1.90	20.76	2.05
G -> R	479,056	-5.90	1.91	3.75	72.22	7.23
G -> N	479,056	-4.76	2.10	3.11	49.29	4.86
G -> D	479,056	-7.01	2.37	3.43	50.64	5.15
G -> C	479,056	-6.00	2.36	2.51	31.73	2.92
G -> Q	479,056	-4.51	2.31	3.39	58.20	5.61
G -> E	479,056	-5.00	2.47	3.61	52.90	5.76
G -> H	479,056	-3.79	2.47	3.37	105.82	5.41
G -> I	479,056	-6.08	3.59	4.52	56.48	5.92
G -> L	479,056	-5.48	2.26	3.35	56.92	5.69
G -> K	479,056	-4.61	2.04	3.40	61.33	6.08
G -> M	479,056	-5.69	2.05	2.77	51.76	4.68
G -> F	479,056	-5.92	2.31	5.09	97.75	10.27
G -> P	479,056	-5.49	5.31	5.01	49.18	4.29
G -> S	479,056	-2.90	2.06	2.32	29.09	2.77
G -> T	479,056	-4.02	3.11	3.76	41.32	4.38
G -> W	479,056	-6.07	2.57	6.59	116.63	13.62
G -> Y	479,056	-6.57	2.43	5.60	105.19	11.37
G -> V	479,056	-5.36	3.65	4.13	46.69	4.67

**Mutation**	**N**	**Min**	**Median**	**Mean**	**Max**	**SD**
H -> A	195,650	-69.93	0.36	0.66	89.98	6.85
H -> R	195,650	-71.06	-0.11	0.47	97.17	6.99
H -> N	195,650	-70.08	0.23	0.60	90.59	6.86
H -> D	195,650	-70.17	0.66	1.11	92.29	6.93
H -> C	195,650	-70.21	0.42	0.75	90.61	6.84
G -> Q	195,650	-70.91	0.04	0.45	90.57	6.86
H -> E	195,650	-69.92	0.32	0.78	90.41	6.90
H -> G	195,650	-69.66	0.92	1.22	91.48	6.91
H -> I	195,650	-71.38	0.21	0.62	91.98	6.93
H -> L	195,650	-71.51	-0.33	-0.17	90.03	6.87
H -> K	195,650	-70.89	-0.18	0.20	90.26	6.87

H -> M	195,650	-71.9	-0.50	-0.34	89.94	6.87
H -> F	195,650	-71.90	-0.39	-0.15	90.87	6.91
H -> P	195,650	-70.87	2.61	2.57	94.28	7.21
H -> S	195,650	-69.80	0.48	0.87	90.33	6.86
H -> T	195,650	-69.92	0.58	0.98	90.60	6.88
H -> W	195,650	-70.51	-0.11	0.69	105.05	7.31
H -> Y	195,650	-71.16	-0.23	0.30	94.39	7.04
H -> V	195,650	-71.04	0.51	0.90	91.16	6.88

**Mutation**	**N**	**Min**	**Median**	**Mean**	**Max**	**SD**
I -> A	288,358	-19.07	2.02	2.04	6.89	1.58
I -> R	288,358	-8.33	1.43	2.82	44.23	3.53
I -> N	288,358	-9.74	2.03	2.06	14.75	1.61
I -> D	288,358	-10.18	2.80	2.86	15.66	2.22
I -> C	288,358	-11.53	1.88	1.90	9.70	1.27
I -> Q	288,358	-9.02	1.61	1.78	14.26	1.61
I -> E	288,358	-8.47	2.10	2.40	13.63	2.11
I -> G	288,358	-38.01	3.06	3.01	8.68	1.75
I -> H	288,358	-8.47	1.62	2.01	59.68	1.74
I -> L	288,358	-8.31	0.06	0.18	18.24	0.63
I -> K	288,358	-8.54	1.20	1.87	22.55	2.10
I -> M	288,358	-9.01	0.05	0.17	14.36	0.73
I -> F	288,358	-7.88	0.83	2.59	56.71	3.84
I -> P	288,358	-3.94	3.95	3.77	19.81	2.46
I -> S	288,358	-12.00	2.49	2.53	10.12	1.88
I -> T	288,358	-5.81	1.73	1.75	6.29	1.30
I -> W	288,358	-7.76	1.54	5.09	68.56	7.02
I -> Y	288,358	-7.82	1.23	3.94	60.89	5.45
I -> V	288,358	-3.35	0.81	0.80	5.84	0.46

**Mutation**	**N**	**Min**	**Median**	**Mean**	**Max**	**SD**
L -> A	687,525	-15.40	1.67	1.90	6.74	1.53
L -> R	687,525	-4.02	1.13	2.27	47.28	3.00
L -> N	687,525	-4.69	1.73	1.85	11.75	1.58
L -> D	687,525	-5.71	2.34	2.64	11.21	2.19
L -> C	687,525	-10.43	1.81	1.89	7.32	1.31
L -> Q	687,525	-4.77	1.35	1.55	12.58	1.43
L -> E	687,525	-4.91	1.70	2.16	12.71	1.98
L -> G	687,525	-28.59	2.61	2.74	8.76	2.17
L -> H	687,525	-4.07	1.46	1.84	56.83	1.63
L -> I	687,525	-4.99	0.78	1.11	37.24	1.13
L -> K	687,525	-4.72	0.93	1.55	14.25	1.77
L -> M	687,525	-4.71	-0.01	0.06	11.34	0.48
L -> F	687,525	-3.36	0.44	1.59	59.52	2.73
L -> P	687,525	-5.84	3.97	3.91	25.50	2.72
L -> S	687,525	-11.16	2.07	2.28	7.96	1.87
L -> T	687,525	-4.10	1.91	2-03	26.20	1.46

L -> W	687,525	-4.22	0.98	3.69	94.86	5.63
L -> Y	687,525	-3.56	0.77	2.87	67.78	4.36
L -> V	687,525	-5.23	1.48	1.66	27.57	1.12

**Mutation**	**N**	**Min**	**Median**	**Mean**	**Max**	**SD**
K -> A	443,672	-9.20	0.50	0.58	6.38	0.64
K -> R	443,672	-4.91	0.05	0.04	14.88	0.56
K -> N	443,672	-5.84	0.46	0.57	7.24	0.76
K -> D	443,672	-6.11	0.79	1.00	11.84	1.18
K -> C	443,672	-8.31	0.71	0.77	6.53	0.60
K -> Q	443,672	-7.25	0.21	0.31	7.39	0.57
K -> E	443,672	-4.88	0.46	0.63	11.52	0.98
K -> G	443,672	-14.87	1.02	1.04	8.01	1.09
K -> H	443,672	-3.82	0.51	0.49	61.28	0.67
K -> I	443,672	-4.16	0.39	0.63	35.30	0.98
K -> L	443,672	-4.84	0.01	0.01	7.98	0.54
K -> M	443,672	-4.90	-0.09	-0.07	6.06	0.59
K -> F	443,672	-4.69	0.07	0.29	52.06	1.32
K -> P	443,672	-5.85	2.52	2.37	26.23	2.25
K -> S	443,672	-8.77	0.62	0.74	7.38	0.79
K -> T	443,672	-3.98	0.80	0.93	27.81	0.83
K -> W	443,672	-4.12	0.26	0.59	66.18	1.67
K -> Y	443,672	-4.59	0.16	0.44	54.17	1.64
K -> V	443,672	-3.82	0.75	0.94	31.87	0.80

**Mutation**	**N**	**Min**	**Median**	**Mean**	**Max**	**SD**
M -> A	150,890	-8.70	1.24	1.56	5.95	1.36
M -> R	150,890	-3.21	0.82	1.59	39.50	2.26
M -> N	150,890	-3.00	1.27	1.61	12.86	1.55
M -> D	150,890	-5.62	1.68	2.22	11.15	2.10
M -> C	150,890	-4.82	1.49	1.58	6.70	1.24
M -> Q	150,890	-3.03	0.95	1.27	13.50	1.31
M -> E	150,890	-3.45	1.16	1.76	13.23	1.81
M -> G	150,890	-12.93	2.04	2.25	8.14	1.98
M -> H	150,890	-2.66	1.16	1.54	71.11	1.57
M -> I	150,890	-2.82	0.76	1.09	21.92	1.13
M -> L	150,890	-3.74	0.19	0.28	18.50	0.54
M -> K	150,890	-3.83	0.65	1.12	17.30	1.39
M -> F	150,890	-2.79	0.42	1.67	63.30	3.19
M -> P	150,890	-4.23	3.65	3.54	22.17	2.74
M -> S	150,890	-5.76	1.48	1.84	6.88	1.66
M -> T	150,890	-2.55	1.58	1.76	17.05	1.41
M -> W	150,890	-3.47	0.66	2.97	63.02	5.14
M -> Y	150,890	-2.86	0.63	2.58	60.99	4.54
K -> V	150,890	-2.63	1.34	1.54	17.09	1.15

Mutation	N	Min	Median	Mean	Max	SD
F -> A	243,115	-17.81	2.78	2.65	7.82	1.83
F -> R	243,115	-6.89	2.32	2.63	42.64	2.48
F -> N	243,115	-10.81	2.89	2.66	7.89	1.93
F -> D	243,115	-11.82	3.70	3.42	15.90	2.47
F -> C	243,115	-15.12	2.73	2.59	8.13	1.67
F -> Q	243,115	-9.97	2.52	2.39	12.01	1.84
F -> E	243,115	-10.29	3.03	2.98	9.80	2.29
F -> G	243,115	-18.09	3.78	3.51	9.51	2.37
F -> H	243,115	-10.29	2.30	2.44	7.33	1.79
F -> I	243,115	-7.69	1.91	2.11	22.15	1.69
F -> L	243,115	-9.17	0.90	1.02	9.06	0.99
F -> K	243,115	-7.88	2.07	2.16	31.35	1.86
F -> M	243,115	-15.07	0.63	0.64	9.94	0.69
F -> P	243,115	-13.37	4.59	4.60	19.47	3.00
F -> S	243,115	-15.27	3.13	3.01	8.87	2.13
F -> T	243,115	-10.59	2.95	2.84	12.85	1.88
F -> W	243,115	-4.22	0.94	1.72	61.29	2.36
F -> Y	243,115	-5.73	0.50	0.80	53.95	1.03
F -> V	243,115	-10.61	2.42	2.46	14.16	1.57

Mutation	N	Min	Median	Mean	Max	SD
P -> A	473,592	-12.14	0.90	0.93	8.73	1.24
P -> R	473,592	-12.74	0.63	1.15	75.06	3.18
P -> N	473,592	-12.52	0.90	1.02	29.74	1.88
P -> D	473,592	-12.94	0.80	1.02	30.51	2.01
P -> C	473,592	-12.50	1.00	1.02	18.36	1.51
P -> Q	473,592	-12.83	0.70	0.90	45.42	2.02
P -> E	473,592	-12.66	0.58	0.84	42.58	2.00
P -> G	473,592	-12.40	1.02	1.10	7.58	1.75
P -> H	473,592	-12.66	0.77	0.96	97.74	1.88
P -> I	473,592	-11.92	0.94	1.08	35.50	1.73
P -> L	473,592	-12.76	0.68	0.81	37.87	1.82
P -> K	473,592	-12.81	0.65	0.95	51.12	2.46
P -> M	473,592	-12.70	0.64	0.72	38.43	1.65
P -> F	473,592	-12.69	0.64	1.39	66.33	3.91
P -> S	473,592	-12.87	0.94	1.01	15.10	1.63
P -> T	473,592	-12.01	1.06	1.19	29.63	1.72
P -> W	473,592	-12.47	0.75	2.00	110.26	5.82
P -> Y	473,592	-12.69	0.71	1.67	83.84	4.69
P -> V	473,592	-11.87	1.12	1.16	31.75	1.48

Mutation	N	Min	Median	Mean	Max	SD
S -> A	602,870	-4.75	-0.01	0.07	4.31	0.69
S -> R	602,870	-5.83	-0.24	0.43	50.00	3.03
S -> N	602,870	-4.00	-0.01	0.29	18.90	1.34
S -> D	602,870	-6.41	0.12	0.49	21.57	1.67

S -> C	602,870	-6.95	0.09	0.25	7.32	0.70
S -> Q	602,870	-3.87	-0.15	0.31	29.12	1.82
S -> E	602,870	-6.17	-0.04	0.43	35.08	1.99
S -> G	602,870	-12.68	0.29	0.29	5.25	0.98
S -> H	602,870	-4.10	0.10	0.35	82.87	1.66
S -> I	602,870	-5.88	0.26	0.66	41.70	1.60
S -> L	602,870	-6.04	-0.18	0.09	24.45	1.62
S -> K	602,870	-4.88	-0.24	0.27	39.81	2.18
S -> M	602,870	-6.00	-0.29	-0.13	22.86	1.50
S -> F	602,870	-6.41	-0.18	0.88	66.38	4.36
S -> P	602,870	-5.74	1.90	1.84	39.19	2.40
S -> T	602,870	-3.39	0.26	0.50	27.75	0.96
S -> W	602,870	-6.07	0.00	1.72	80.88	6.57
S -> Y	602,870	-5.47	-0.11	1.22	69.93	5.22
S -> V	602,870	-4.81	0.48	0.74	32.32	1.22

**Mutation**	**N**	**Min**	**Median**	**Mean**	**Max**	**SD**
T -> A	367,715	-8.36	0.15	0.26	6.49	0.81
T -> R	367,715	-5.65	-0.18	0.55	42.29	2.76
T -> N	367,715	-4.66	0.11	0.34	18.40	1.03
T -> D	367,715	-6.71	0.31	0.69	17.52	1.52
T -> C	367,715	-6.15	0.20	0.35	12.15	0.73
T -> Q	367,715	-4.70	-0.08	0.30	17.66	1.41
T -> E	367,715	-5.75	0.06	0.54	19.16	1.68
T -> G	367,715	-10.48	0.55	0.71	8.03	1.22
T -> H	367,715	-4.64	0.11	0.34	64.18	1.27
T -> I	367,715	-4.93	-0.07	0.05	13.42	1.16
T -> L	367,715	-8.72	-0.28	-0.22	18.86	1.10
T -> K	367,715	-4.98	-0.21	0.27	26.35	1.83
T -> M	367,715	-5.33	-0.37	-0.29	23.33	1.16
T -> F	367,715	-6.10	-0.19	0.81	47.25	3.54
T -> P	367,715	-10.94	1.88	1.84	28.18	2.25
T -> S	367,715	-6.30	0.21	0.34	12.91	0.77
T -> W	367,715	-5.32	0.03	1.85	68.73	5.86
T -> Y	367,715	-5.24	-0.10	1.29	58.07	4.58
T -> V	367,715	-4.31	0.11	0.23	9.93	0.87

**Mutation**	**N**	**Min**	**Median**	**Mean**	**Max**	**SD**
W -> A	79,980	-20.50	2.49	2.63	9.04	2.11
W -> R	79,980	-13.52	2.12	2.35	40.6	2.27
W -> N	79,980	-20.10	2.64	2.71	9.64	2.24
W -> D	79,980	-17.26	3.24	3.30	11.02	2.72
W -> C	79,980	-21.24	2.56	2.68	9.13	2.01
W -> Q	79,980	-18.94	2.32	2.41	9.78	2.11
W -> E	79,980	-16.67	2.67	2.87	10.34	2.51
W -> G	79,980	-21.21	3.43	3.45	10.40	2.59
W -> H	79,980	-17.26	2.13	2.44	9.04	2.06

W -> I	79,980	-18.21	1.86	2.19	21.14	1.97
W -> L	79,980	-21.84	0.99	1.32	26.89	1.46
W -> K	79,980	-15.91	1.96	2.21	52.96	2.14
W -> M	79,980	-20.90	0.73	0.97	39.76	1.19
W -> F	79,980	-12.42	0.51	0.80	32.07	1.23
W -> P	79,980	-19.27	4.16	4.40	20.49	3.25
W -> S	79,980	-20.87	2.93	3.01	9.71	2.39
W -> T	79,980	-18.63	2.76	2.90	14.86	2.19
W -> Y	79,980	-9.68	0.82	1.21	33.65	1.64
W -> V	79,980	-18.73	2.30	2.53	15.94	1.89

**Mutation**	**N**	**Min**	**Median**	**Mean**	**Max**	**SD**
Y -> A	182,982	-27.99	1.97	2.14	8.70	1.64
Y -> R	182,982	-15.17	1.53	1.86	70.19	1.94
Y -> N	182,982	-19.50	2.00	2.16	8.76	1.76
Y -> D	182,982	-18.84	2.71	2.86	10.57	2.28
Y -> C	182,982	-22.0	2.03	2.14	8.24	1.51
Y -> Q	182,982	-17.14	1.77	1.94	21.26	1.70
Y -> E	182,982	-14.93	2.15	2.42	10.65	2.11
Y -> G	182,982	-45.17	2.97	2.99	10.78	2.13
Y -> H	182,982	-18.37	1.66	1.93	8.25	1.59
Y -> I	182,982	-16.20	1.38	1.76	22.81	1.66
Y -> L	182,982	-16.07	0.42	0.74	23.52	1.05
Y -> K	182,982	-14.61	1.29	1.58	23.41	1.57
Y -> M	182,982	-18.37	0.26	0.48	11.83	0.87
Y -> F	182,982	-4.89	-0.06	0.03	20.19	0.61
Y -> P	182,982	-22.10	4.12	4.18	18.47	2.93
Y -> S	182,982	-23.20	2.46	2.54	9.50	1.93
Y -> T	182,982	-18.45	2.27	2.43	11.15	1.75
Y -> W	182,982	-3.78	0.45	1.07	72.25	1.89
Y -> V	182,982	-18.76	1.79	2.04	11.22	1.48

**Mutation**	**N**	**Min**	**Median**	**Mean**	**Max**	**SD**
V -> A	405,338	-9.43	0.94	1.19	8.46	1.32
V -> R	405,338	-5.75	0.42	2.49	53.31	4.37
V -> N	405,338	-7.43	0.99	1.33	14.91	1.51
V -> D	405,338	-9.38	1.44	2.02	16.24	2.12
V -> C	405,338	-14.27	0.91	1.08	9.76	1.01
V -> Q	405,338	-5.23	0.60	1.37	18.34	2.02
V -> E	405,338	-4.63	0.92	1.83	18.22	2.36
V -> G	405,338	-20.56	1.93	2.10	9.38	2.04
V -> H	405,338	-5.75	0.73	1.36	77.30	1.78
V -> I	405,338	-4.13	-0.27	-0.19	26.70	0.64
V -> L	405,338	-8.86	-0.18	0.04	19.43	1.03
V -> K	405,338	-6.36	0.28	1.59	36.85	2.80
V -> M	405,338	-7.85	-0.17	0.12	17.57	1.29
V -> F	405,338	-5.78	0.17	2.57	56.87	4.74

V -> P	405,338	-12.30	2.87	2.82	26.72	2.37
V -> S	405,338	-14.8	1.37	1.68	9.24	1.66
V -> T	405,338	-4.96	0.71	0.93	11.88	1.04
V -> W	405,338	-4.34	0.61	5.04	75.17	8.31
V -> Y	405,338	-5.65	0.38	3.76	62.93	6.34
